# Effect of SiO_2_ Particles on the Relaxation Dynamics of Epoxidized Natural Rubber (ENR) in the Melt State by Time-Resolved Mechanical Spectroscopy

**DOI:** 10.3390/polym13020276

**Published:** 2021-01-15

**Authors:** Rossella Arrigo, Leno Mascia, Jane Clarke, Giulio Malucelli

**Affiliations:** 1 Department of Applied Science and Technology and local INSTM Unit, Politecnico di Torino, Viale Teresa Michel 5, 15121 Alessandria, Italy; giulio.malucelli@polito.it; 2 Department of Materials, Loughborough University, Loughborough LE11 3TU, UK; l.mascia@lboro.ac.uk (L.M.); J.Clarke@lboro.ac.uk (J.C.)

**Keywords:** epoxidized natural rubber, ENR, silica nanocomposites, time-resolved mechanical spectroscopy, rheological behavior, gelation

## Abstract

The rheological behavior of an epoxidized natural rubber (ENR) nanocomposite containing 10 wt.% of silica particles was examined by time-resolved mechanical spectroscopy (TRMS), exploiting the unique capability of this technique for monitoring the time-dependent characteristics of unstable polymer melts. The resulting storage modulus curve has revealed a progressive evolution of the elastic component of the composite, associated with slower relaxations of the ENR macromolecular chains. Two major events were identified and quantified: one is associated with the absorption of the epoxidized rubber macromolecules onto the silica surface, which imposes further restrictions on the motions of the chains within the polymer phase; the second is related to gelation and the subsequent changes in rheological behavior resulting from the simultaneous occurrence cross-linking and chain scission reactions within the ENR matrix. These were quantified using two parameters related to changes in the storage and loss modulus components.

## 1. Introduction

The reinforcement of elastomers through the introduction of inorganic fillers is not only widely used for large scale manufacture of rubber products but also to formulate smart materials tailored to meet specific requirements in different fields of application [1,2,3]. The effective improvement of the material final properties achieved in elastomer-based composites depends on several factors, including the geometric feature dimensions of the reinforcing fillers [4], the extent of dispersion within the host matrix and their possible orientation [5], as well as their chemical nature, governing the possible establishment of strong polymer–filler interactions and, hence, the characteristics of the interfacial region [6].

Although silica and carbon black represent the two class of fillers that are most widely used to produce elastomeric composites, over the last few decades, other inorganic nanofillers, such as carbon-based nanostructures [7,8] and phyllosilicates [9], have attracted a steadily increasing interest, due to the possibility to achieve similar property enhancements at significantly lower filler content with respect to the traditional micro-particles [10,11].

The utilization of natural rubber (NR) can be further extended by introducing reactive groups that may favor the compatibilization of the elastomer with other polymers or inorganic fillers, achievable by widening the possibilities of chemical reactions and increased physical interactions resulting from the increase in polarity. Epoxidation of NR as a route to achieve these objectives has been extensively reported in the literature [12,13]. Due to the reactivity of epoxy groups and the high polarity of OH groups, epoxidized natural rubber (ENR) is widely used for applications requiring strong adhesion and to enhance the interfacial adhesion with silica filler and nanoparticles to increase the reinforcement efficiency and related performance of products, such as tires [14,15,16].

Despite the wide use of filler-containing rubbers at the industrial level and the extensive research carried out over the years, the field is still open to challenges to achieve highly effective reinforcement in soft matrix composites and nanocomposites owing to the alteration of the dynamics of the polymer chains [17]. The established effects of polymer–filler interaction and the elastic properties inherent to these systems, alongside modifications of the polymer network, will have a substantial effect on the viscoelastic behavior, including the variation of both linear and non-linear dynamic response of the composite material [18]. In particular, the introduction of silica nanoparticles into rubber matrices strongly affects the linear viscoelastic behavior, causing a progressive increase in the storage and loss moduli values as a function of the particle loading [19] and a disappearance of the terminal flow behavior, as a consequence of the incomplete relaxation of the filler network and the restricted motion of the macromolecular chains [20]. In this context, Song et al. [21] investigated the rheological behavior of natural rubber-based composites containing different amounts of precipitated silica particles, demonstrating a liquid-to-solid transition associated with the networking of nanoparticles interconnected by rubber chain bridges.

Therefore, a good understanding of the viscoelastic behavior of elastomer-based composites is required to gain a deeper knowledge of the reinforcement mechanisms for the systems and to fully exploit the potential of these materials. This is particularly important in some specific applications, such as tires, where the viscoelastic properties of the unfilled elastomer have to be preserved in order to achieve a high performance in terms of wet grip and rolling resistance [22].

Generally, the viscoelastic behavior of polymer-based composites is evaluated through rheological measurements as a way of obtaining important information about the structure of the material and attenuation of polymer chain relaxations resulting from the introduction of fillers [23]. In this context, it is particularly relevant the evaluation of the material rheological response recorded at very low frequencies, where the dynamics of a large portion of material relaxing slowly are probed [24]. Nonetheless, conventional rheological measurements performed at low-frequency experience limitations related to the duration of the experimental test, which may bring about molecular and microstructure changes [25,26]. Accordingly, if the polymer microstructure changes during the test in a time interval exceeding the relaxation time of the polymer, the mobility of the macromolecules and their relaxation dynamics are considerably altered. Therefore, the rheological data collected using conventional frequency sweep tests are not always reliable [27]. It has been shown that an accurate characterization of the rheological behavior of polymers experiencing time-related mutations can be achieved using time-resolved mechanical spectroscopy (TRMS) [28,29]. In these tests, the material is subjected to several frequency-sweep tests, and the collected data are presented as a function of the acquisition time. Through an extrapolation procedure, it is possible to estimate the time-zero rheological functions, representing the “true” rheological response of the material. This method makes it possible to decouple the effects of time and changes in macromolecular structure on the recorded rheological response of polymer-based systems in transient states of evolution. A similar approach is also usually exploited for characterizing the thermal and thermo-oxidative degradation of polymers and blends [30].

In a previous work, we have explored the potential of a TRMS analysis to evaluate the time-dependent rheological behavior of an epoxidized natural rubber containing 25 mol% of epoxy groups, which experienced structural changes and gelation in the molten state [31]. The obtained results have indicated an alteration of the polymer relaxation dynamics through the formation of a highly elastic network due to the occurrence of thermal-activated reactions associated with the formation of crosslinks.

In this later work, we have used a similar approach to obtain an accurate characterization of the rheological behavior of an ENR-based composite, containing 10 wt.% of silica particles. To this end, the relaxation spectrum of the material was obtained to evaluate the effect of the embedded particles on the dynamics of ENR macromolecules. TRMS was also used to assess the possibility of utilizing the changes in rheological parameters occurring by the thermal treatment received by the melt during the test as a method to probe the effect of silica particles on cross-linking and chain scission reactions in the matrix.

## 2. Materials and Methods

### 2.1. Materials

**Figure 1:**
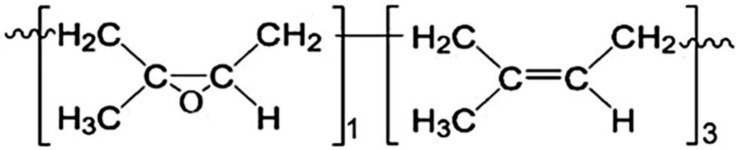
Chemical formula of epoxidized natural rubber (ENR)25.

An epoxidized natural rubber (ENR25) containing 25 mol% of epoxy groups (Epoxyprene 25, manufactured by Muang Mai Guthrie Public Limited Company of Muang, Thailand and donated by Tun Abdul Razak Research Centre) was employed as matrix. ENR25 has Mooney viscosity in the range 70–100 MU (corresponding to an Mn (average) in the region of 80 kDa) and a glass transition temperature of −45 °C. The chemical structure of ENR 25 is shown in Figure 1 [32]:

It should be noted that ENR25 retains the *Cis*-polyisoprene configuration of natural rubber, which is retained also by the 25% epoxidized units. Accordingly, ENR retains the unique strain hardening characteristics of natural rubber up to quite high levels of epoxidation [33].

Silica nanoparticles (SiO_2_), Sidistar T120 (Elkem, Oslo, Norway), with specific surface area (BET) in the range 18–25 m^2^/g and 150 nm median particle size were used as filler.

### 2.2. Processing

ENR + SiO_2_ composites containing 10 wt.% of silica particles were prepared using a Brabender-Plastograph mixer operating at 90 °C and 100 rpm for 10 min. An amount of 10 wt.% was selected as optimal silica particles loading to achieve appreciable modifications of the rheological behavior of the unfilled ENR, without compromising the obtainment of a homogeneous dispersion of silica particles within the host matrix. Furthermore, we chose relatively coarse nanoparticles (125 nm) and a low silica content to avoid interferences from particle–particle interactions (percolation and flocculation phenomena), which would have confounded the interpretation of the dynamic relaxations of the matrix.

Unfilled ENR was subjected to the same processing operation and conditions. Specimens for the rheological characterization were obtained by compression molding, using a laboratory press (Collin Teach Line 200T), working at 90 °C with an applied pressure of 100 bar for 2 min.

### 2.3. Characterizations

Rheological measurements were performed using an ARES (TA Instrument, New Castle, DE, USA) strain-controlled rheometer with a parallel plate geometry and a plate diameter of 25 mm. Preliminary time sweep tests were performed at 180 °C at a wide range of frequencies.

The testing temperature was selected in order to achieve a full plasticization of the polymer matrix, as well as to speed up the possible modifications occurring in the rubber during TRMS tests. Small amplitude oscillatory shear (SAOS) measurements were performed at 180 °C with frequency scans corresponding to angular velocity in the range 0.1–100 rad/s. The strain amplitude was fixed at 0.1% to ensure the recorded response was within the linear viscoelastic region. Large amplitude oscillatory shear (LAOS) behavior was characterized through strain sweep measurements performed at 180 °C and ω = 1 rad/s. Time-resolved mechanical spectroscopy (TRMS) tests were carried out in air at 180 °C, subjecting the samples to 10 repeated frequency sweeps over a range of angular velocity, varying from 10^−1^ to 10^2^ rad/s. Both storage (G’) and loss (G”) modulus were recorded during the measurements within the linear viscoelastic region. In all the measurements, the gap between the plates was set to 1 mm.

The morphology of the ENR + SiO_2_ composite was examined using a LEO-1450VP (Zeiss, Oberkochen, Germany) scanning electron microscope (SEM) (beam voltage: 20 kV). The observations were performed on the cross-sections the samples, fractured in liquid nitrogen. The fracture surfaces were coated with a thin gold layer.

ATR-FTIR spectra were collected using a Perkin Elmer Spectrum 100 spectrometer (Shelton, CT, USA) equipped with an attenuated total reflection (ATR) diamond probe. FTIR spectra were recorded at wavelengths from 700 to 4000 cm^−1^ with 4 cm^−1^ resolution and collecting 16 scans. Quantitative estimates of the reduction in epoxy groups were made on ENR and ENR + SiO_2_, before and after TRMS measurements, normalizing the height of the peak associated with epoxy groups (at 875 cm^−1^) to that of the signal related to the methyl asymmetrical stretching vibrations (at 2962 cm^−1^).

## 3. Results and Discussion

### 3.1. Linear Rheological Behavior

**Figure 2:**
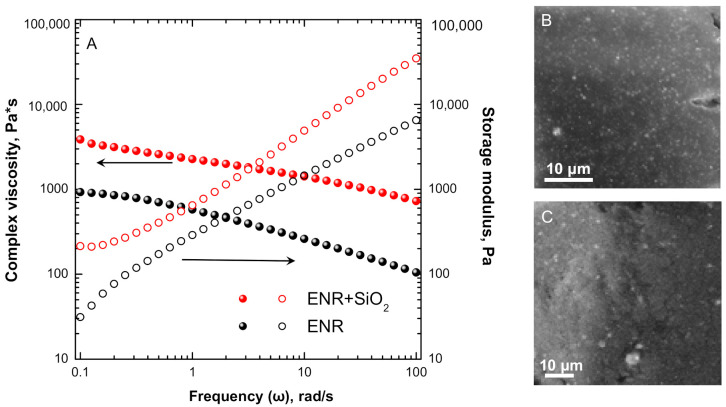
(**A**) Complex viscosity (full symbols) and storage modulus (empty symbols) and (**B**,**C**) SEM micrographs for ENR + SiO_2_ composite at different magnifications.

The data obtained from frequency sweep measurements, performed to ascertain the linearity of the rheological behavior of the samples, are shown Figure 2A. Here are compared the variations of the complex viscosity and the dynamic storage modulus as a function of frequency (expressed as angular velocity) for the composite system to those of the pristine (filler-free) elastomer. The trendlines display the typical reinforcement effect brought about by the incorporation of silica particles and an approximate power law behavior for both rheological parameters. Moreover, a peculiar feature of the data for the storage modulus is the behavior in the low-frequency region (0.1–0.2 rad/s), where the two systems exhibit an opposite trend, while the viscosity for the composite melt does not display the tendency for the usual Newtonian plateau. This behavior could be attributed to a modification of the relaxation dynamics of ENR macromolecules, due to the establishment of polymer–filler and filler–filler interactions, able to restrain the long-range motion of ENR macromolecular chains [34]. The SEM micrographs reported in Figure 2B,C show that the embedded particles are homogeneously dispersed within ENR, notwithstanding the presence of few micron-sized aggregates.

To further investigate the effect of the embedded particles on the relaxation dynamics of ENR macromolecules, the continuous relaxation spectra for both investigated systems were obtained following the calculation procedure proposed by Honerkamp and Weese [35], which is founded on the response of an infinite number of Maxwell models placed in parallel [36]:

(1) $$G^{\prime }\left(\omega \right)=\int_{-\infty }^{+\infty }H\left(\lambda \right)\frac{\omega^{2}\lambda^{2}}{1+\omega^{2}\lambda^{2}}dln\lambda$$

(2) $$G\prime \prime \left(\omega \right)=\int_{-\infty }^{+\infty }H\left(\lambda \right)\frac{\omega \lambda }{1+\omega^{2}\lambda^{2}}dln\lambda$$

where *G*’(*ω*) and *G*”(*ω*) are storage and loss moduli measured through frequency sweep tests, *H*(*λ*) is the relaxation time spectrum, and *λ* is the relaxation time.

**Figure 3:**
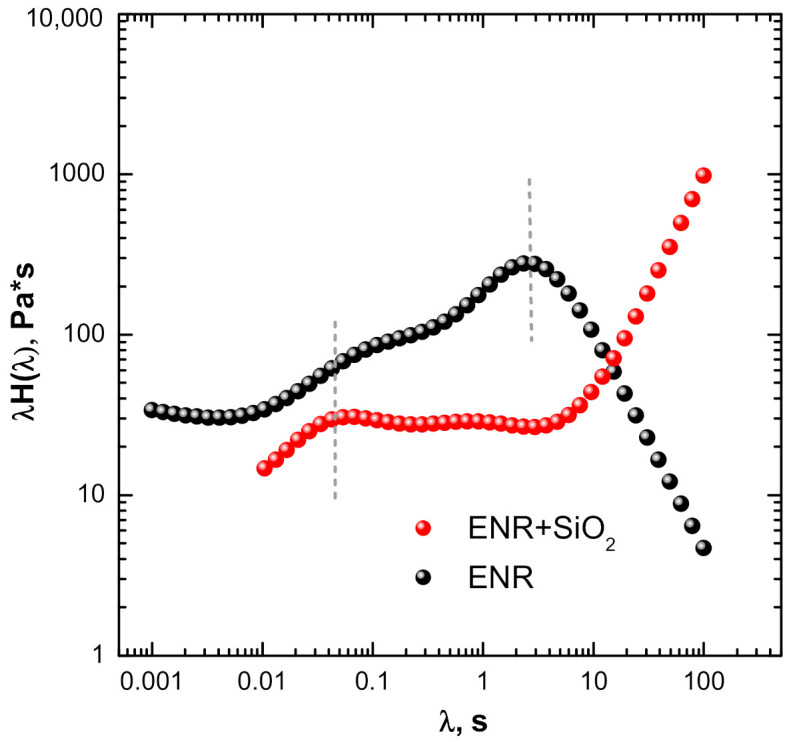
“Weighted” relaxation spectra for ENR and ENR + SiO_2_ composite.

The plots in Figure 3 show the “weighted” relaxation spectra for both pristine (unfilled) and the ENR + SiO_2_ composite. This type of plot amplifies the contribution of slow processes, thereby making more evident the relaxation of macromolecular chains at the polymer–filler interface [37]. The pristine (unfilled) ENR shows a distinct peak at relaxation time in the region of 5 s and a shoulder at shorter relaxation times, which is indicative of the existence of two molecular populations relaxing over different time scales. The occurrence of a sharp decrease in the relaxation time function at longer times (>100 s) suggests that ENR macromolecules can fully relax at the upper end of the experimental time. In contrast, the trendline for the relaxation spectrum of the ENR+ SiO_2_ composite displays a large upward shift in the peak related to the main relaxation mode of ENR chains (λ in the range 1–100 s), while maintaining the fast relaxations at the low end of the scale of relaxation times. This behavior can be ascribed to the presence of substantial matrix/polymer interphases exhibiting highly impeded relaxations relative to the matrix. Such morphological features are well known in the field of reinforced elastomers and are associated with the formation of both interfacial chemical bonds and interphase domains of higher cross-linking density. Recent computer modeling studies have shown how double bond interactions from a diene elastomer (*cis*- and *trans-* polybutadiene) with silica nanoparticles are sufficient to bring about the formation of an interphase layer [38].

For the system of this study, the effects may be enhanced by the immobilization of the ENR chains on the silica surface by the strong H-bonds arising from the presence of adjacent OH groups produced by the opening of the oxirane ring, which is catalyzed by the acidic nature of the SiOH groups [33].

### 3.2. Non-Linear Rheological Behavior

**Figure 4:**
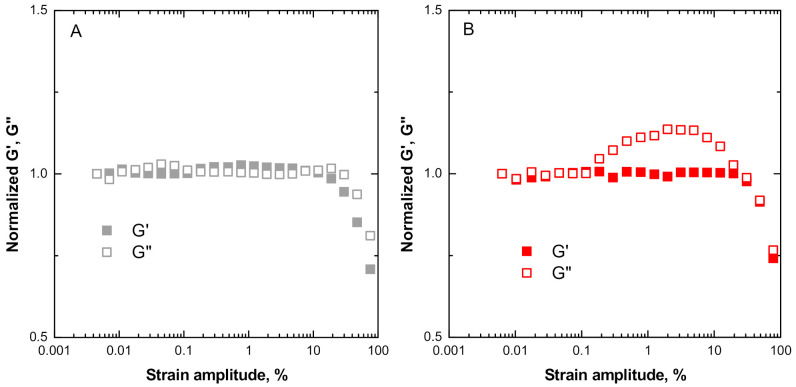
Large amplitude oscillatory shear (LAOS) rheological data for (**A**) pristine ENR and (**B**) ENR + SiO_2_ composite.

The effect of the incorporation of SiO_2_ particles in the ENR matrix on possible non-linearities in the rheological behavior was evaluated using strain sweep runs and performing the tests at large oscillatory deformations. The degree on non-linearity was obtained by normalizing the recorded *G*’ and *G*” relative to the values at low strain (i.e., within the linear viscoelastic region). A ratio greater than 1 for *G*’ is indicative of strain-hardening melt elasticity, while for *G*” denotes a strain-thickening viscous behavior. The traces in Figure 4 show that pristine ENR exhibits the typical pseudoplastic behavior of polymer melts, characterized by a plateau in the low strain amplitude region, followed by a decrease in both viscous and elastic components at some critical strain amplitude (marking the transition from linear to non-linear viscoelastic regime). The ENR + SiO_2_ composite, on the other hand, displays a distinct strain-thickening viscous behavior over the range 0.2–11% strain, with a maximum in the loss modulus variation at around 6% strain. This feature may be considered as an amplified downward shift of the shallow peak observed for pristine ENR at around 20% strain amplitude.

Strain-softening rheological phenomena are well known for elastomer-based composites and have been associated with a decrease in the energy dissipation capability, related to the destruction of the filler network at high-strain amplitude values [39,40] and with a reduction in the entanglement density at high-strain amplitudes [41].

The mild-strain softening behavior of the pristine ENR system of this study can be attributed to the breaking of physical cross-links, possibly H-bond types, between OH and oxirane groups along the molecular chains. These are not so intense owing to the lack of order in the configuration of the chains in the melt state. For the case of ENR + SiO_2_ composite, on the other hand, the observed “strong” strain-thickening viscous behavior occurs in a time scale corresponding to the relaxation of polymer chain segments, which has been identified in the relaxation spectra (Figure 3) and can be attributed to a retardation of the relaxations of ENR chains resulting from the establishment of polymer–filler interactions at the interface. The softening behavior at high strain, on the other hand, can be expected to arise from the breaking of strong physical bonds (H-bonds) between the polar oxirane and hydroxyl groups in the ENR matrix and silanol groups on the surface of the SiO_2_ nanoparticles. It is instructive to note also the lack of strain-hardening effects for the elastic component (*G*’) for both ENR and ENR + SiO_2_, which can be expected from the inability of the polymer chains to appreciably increase the intermolecular attractions during uncoiling.

### 3.3. Time-Resolved Mechanical Spectroscopy of ENR/SiO_2_ Composite

#### 3.3.1. Evolution of the Dynamic Rheological Parameters of ENR + SiO_2_ Composite

**Figure 5:**
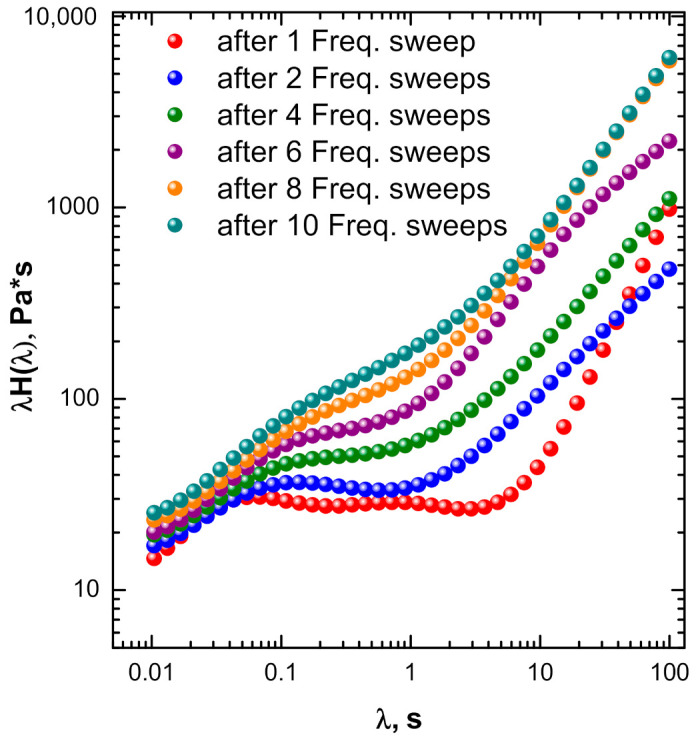
Weighted relaxation spectra for ENR + SiO_2_ system subjected to subsequent frequency sweep measurements.

To gain an insight into the evolution of the material microstructure in the melt state and to evaluate the possible influence of the interaction phenomena between silica and ENR on the relaxation kinetics of the latter, the material was subjected to different successive frequency sweep measurements in order to obtain the weighted relaxation spectra. Looking at the resulting spectra, shown in Figure 5, it is noted that the tail appearing at long relaxation time gradually increases alongside a broadening of the incomplete peak related to the relaxation of ENR macromolecules. These features indicate a progressive elastic dominant behavior evidenced by the appearance of slower relaxation modes of the ENR matrix.

**Figure 6:**
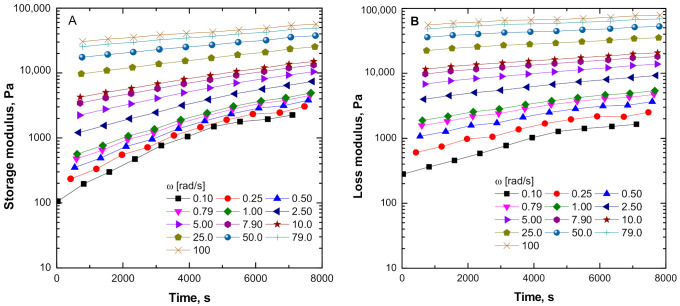
Time-resolved mechanical spectroscopy (TRMS) sweeps for ENR + SiO_2_ at 180 °C: (**A**) storage modulus and (**B**) loss modulus.

To further inspect the phenomena relative to the observed progressive restriction of the ENR relaxation dynamics, the data of the consecutive frequency sweep measurements were re-arranged by applying TRMS procedure, from which plots of the variation of G’ and G” with time reported were made. These are presented in Figure 6A,B and show that both rheometric parameters exhibit an increasing trend with thermal treatment time, which is more pronounced at low frequencies in correspondence to the rheological response of a large portion of the macromolecules.

To gain more detailed insights in the effects of structural changes of the ENR + SiO_2_ composite on its viscoelastic behavior, the respective isochronal modulus values were calculated as the intercept between a vertical line at a specific time t and the curves interpolating the experimental data. Furthermore, the modulus values at time zero were also estimated.

**Figure 7:**
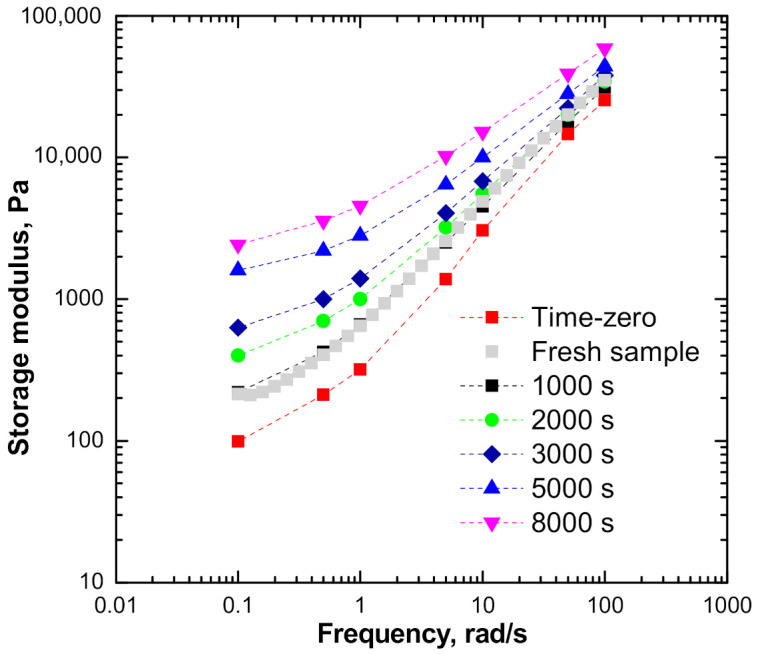
Isochronal storage modulus for ENR + SiO_2_, collected at different times during TRMS tests.

Figure 7 shows the curves of the isochronal storage modulus calculated at different times during TRMS measurements, alongside the calculated G’ value at time zero. The results of single-frequency measurements for ω values over the range 0.1–100 rad/s, performed on a fresh sample of ENR, are also shown for comparison and to illustrate the greater wealth of information obtained using the TRMS procedure.

The TRMS data clearly show not only the enhanced the elastic behavior acquired by the melt with increasing the exposure time to high temperature, but also the vanishing effect of the thermal treatment at much higher frequencies, where the fast relaxations are probed. These two effects are not revealed by conventional single-frequency runs.

#### 3.3.2. Insights into Phenomena Occurring During TRMS

The data collected in the TRMS sweeps for the ENR + SiO_2_ composite are indicative of the possible occurrence of two concurrent phenomena, involving the combined effect of the embedded silica particles and the ENR network evolution, which give rise to a progressive magnification of the elastic rheological behavior.

**Figure 8:**
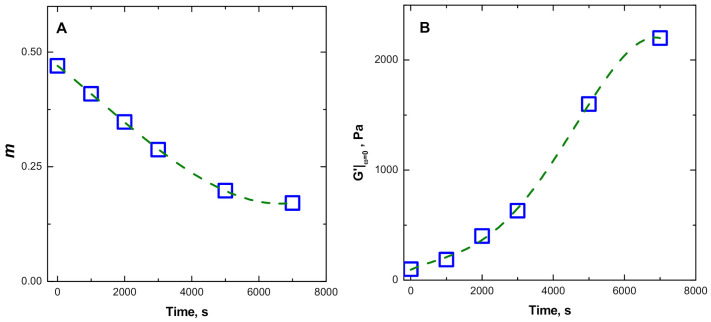
(**A**) Gradient (*m*) of the G’ curves in the terminal region and (**B**) static modulus (*G*’|*ω* = 0) for the for the ENR + SiO_2_ melt.

The values of the gradient of the log–log plot of G’ curves in the terminal region (*m*) and those for the “static” elastic modulus (G’ extrapolated at zero frequency, G’|ω = 0) were calculated and plotted as a function of time in Figure 7. The apparent flattening tendency of the evolution curves for the two factors *m* and G’|ω = 0, which is observed at long thermal treatment times in Figure 8A,B, suggests that a possible stable state may be reached at long thermal treatment times.

In this respect, the downward trend of the gradient m observed in Figure 8A can be taken as indicative of a gradual slowing down of the relaxation kinetics of the ENR macromolecules, since the decrease in the slope of the storage modulus curve in the low-frequency region is often associated with the restriction of the polymer chain motion, resulting from the presence of strong interfacial bonding and highly dispersed particles.

**Figure 9:**
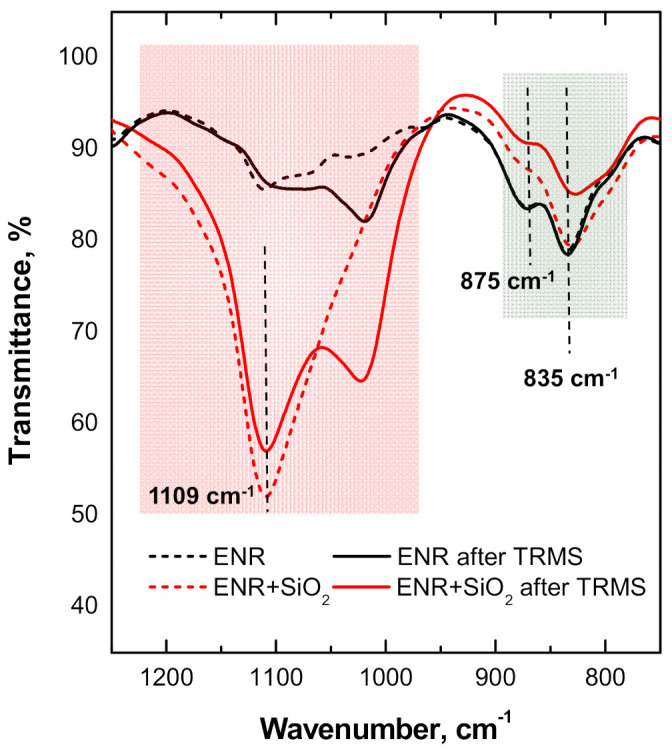
ATR-FTIR spectra for pristine (unfilled) ENR and ENR + SiO_2_ composite before and after TRMS, in the range 1250–750 cm^−1^.

To support the occurrence of strong interactions between ENR macromolecules and the embedded particles, and the possible formation of hydrogen bonds involving OH groups onto silica surface and oxirane rings of the matrix, ATR-FTIR spectra were collected for the unfilled ENR and the ENR + SiO_2_ composite, before and after the thermal treatment (see Figure 9). The introduction of the SiO_2_ particles caused a reduction in the absorption of the peak at 835 cm^−1^ for the double bonds C=CH of ENR [42,43], suggesting a catalytic effect exerted by the silica particles through hydrogen bonding interactions at the interface [44]. At the same time, the unfilled ENR does not show any change in the positioning of the peak at 875 cm^−1^, associated with the epoxy functionality of the matrix, whereas it shifts significantly downwards (by about 40%) in the ENR + SiO_2_ composite subjected to TRMS. This is indicative of the occurrence of ring opening reactions of the epoxy groups during the thermal treatment and strong interactions between silica particles and ENR.

**Figure 10:**
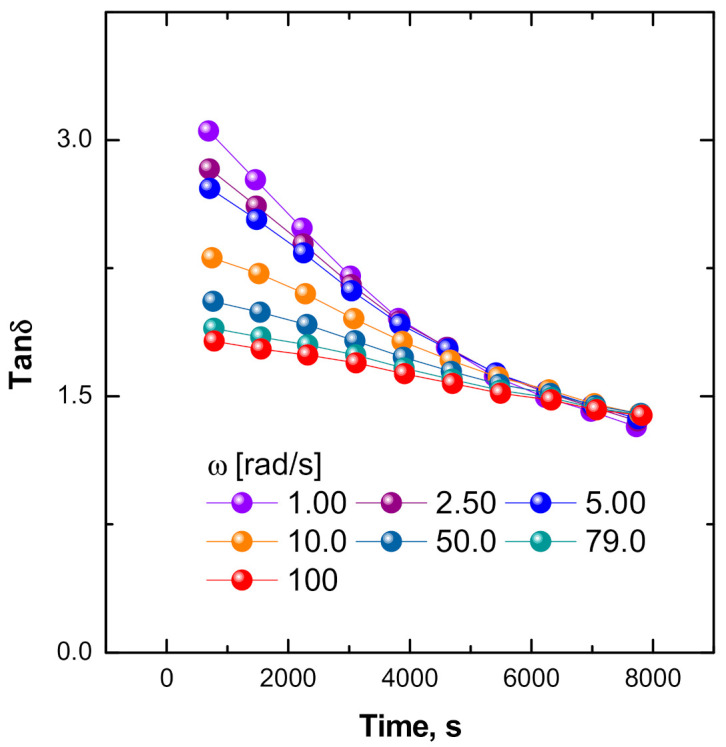
Multi-frequency plot for the variation of tan δ as a function of time for ENR + SiO_2_ system.

In this context, it should be noted that we have previously reported the occurrence of thermal-induced gelation reactions (cross-linking) for the same ENR in the pristine state (unfilled) during TRMS measurements performed at the same temperature [31]. The observed growth trend of the calculated values for static modulus (see Figure 8B) indicates the occurrence of gelation phenomena with a concurrent increase in the cross-linking degree of the ENR matrix. The Winter–Chambon criterion [45] was used to evaluate the critical time for gelation in the ENR + SiO_2_ composite. This method allows the gel time of the system to be identified as the multi-frequency intersection of the G’(ω) and G”(ω) functions. The analysis stems from the approximate power law relationship exhibited by the variation of G’(ω) and G”(ω) with a common relaxation exponent so that the ratio (tan δ) becomes frequency independent and converges and intersects the curves at the gel point, when plotted against time. The multi-frequency plots of tan δ against time for the ENR + SiO_2_ system are presented in Figure 10, from which the intersection point (the gel time) is identified at around 6500 s. For comparison, the measurements performed under the same testing conditions for the ENR sample (matrix component of the ENR + SiO_2_ composite of the present study) the gel point appears at around 1500 s [31]. The estimated increase for the time required to reach the gelation for the composite system can be expected from a possible acceleration of chain scission reactions in ENR the matrix, due to the acidic nature of the SiOH groups on the surface of the silica particles [46,47]. These exert a catalytic effect on the opening of the oxirane groups, which brings about the cleavage of the -C-C- bond at the contiguous -CHOH-CHOH- groups [48].

A discrepancy between the two systems is observed with respect to the values of tan δ at the intersection (tan δ(i)) of G’(ω) and G”(ω) curves, respectively, equal to ~1.5 for ENR + SiO_2_ and ~1.25 for ENR, which are higher than the estimated theoretical value of 1.0. [48].

Values of tan δ(i) greater than the theoretical values are widely reported in the literature and are associated with deviations of the relaxation exponent n from the theoretical value of 0.5 [49]. Conversely, tan δ(i) values substantially below 1.0 have been found for peroxide-cured elastomer systems, where the predominance of rapid cross-linking reactions leads to a very large increase in G’ relative to G’’ [50].

#### 3.3.3. Rheometric Analysis of the Relative Curing Kinetics of ENR + SiO_2_ Composite

For some polymer systems, the rate of the crosslinking reactions by rheometric measurements under isothermal conditions has been determined from measurements of the storage modulus at fixed frequency $G_{t}^{\prime }$ up to the final plateau $G_{\infty }^{\prime }$ using the expression [48,51]:

(3) $$\beta =\frac{G_{t}^{\prime }-G_{0}^{\prime }}{G_{\infty }^{\prime }-G_{0}^{\prime }}$$

where *β* is the extent of reaction, and $G_{0}^{\prime }$ is the value for the storage modulus at time zero.

This rheological analysis, however, has intrinsic limitations, as it does not consider the effects of frequency on the modulus values used in the calculation, and therefore, it only probes changes for specific relaxations within the network. Additional uncertainties would occur in the case of composites, as the measured rheological parameter $G_{t}^{\prime }$ is affected not only by the degree of cross-linking in the matrix, but also by the reinforcement induced by the filler. Furthermore, the application of this analysis to the rheological data of the present work is also prevented by the lack of data for the fully cross-linked state. Nonetheless, in the absence of interferences from the presence of silica particles in the composite system, the values for $(G_{t}^{\prime }-\;G_{0}^{\prime })\;$ are expected to remain invariant, and therefore, a normalized extent cross-linking parameter *β* for ENR + SiO_2_ relative to the pristine ENR makes it possible to follow the progress of the reactions and identify deviations arising from matrix/filler interactions. For a more precise analysis, the treatment would also have to take into consideration the relative changes for the loss modulus parameters, so that two parameters, denoted as *β*’ and *β*’’, can be defined as follows:

(4) $$\beta \prime =\frac{(G_{t}^{\prime }-\;G_{0}^{\prime })\left[\mathrm{ENR}+{\mathrm{SiO}}_{2}\right]}{(G_{t}^{\prime }-\;G_{0}^{\prime })\left[\mathrm{ENR}\right]}\;\mathrm{and}\;\beta \prime \prime =\frac{(G_{t}^{\prime \prime }-\;G_{0}^{\prime \prime })\left[\mathrm{ENR}+{\mathrm{SiO}}_{2}\right]}{(G_{t}^{\prime \prime }-\;G_{0}^{\prime \prime })\left[\mathrm{ENR}\right]}$$

Irrespective of inherent limitations, arising from the effects of frequency on the two viscoelastic parameters, tentative plots of calculated values of *β*’ and *β*’’ against “thermal treatment time”, using dynamic modulus values at 0.1 rad/s and 100 rad/s, are shown in Figure 10 respectively, in order to probe the changes for slow relaxations, involving the entire network and the fast relaxations for small components of the network.

**Figure 11:**
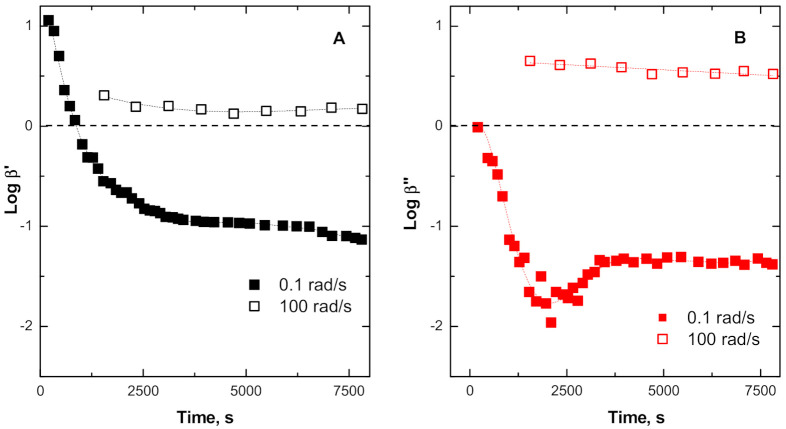
Plots of β’ and β’’ as a function of thermal treatment time.

The plots in Figure 11A,B show that the effect of the silica reinforcement for the fast relaxations (i.e., at ω = 100 rad/s) is not appreciably affected by the thermal treatment for both G’ and G” components. In other words, any resulting increase in cross-linking density or molecular scission degradation takes place at chain length greater than their inherent relaxations. The slow relaxations (associated with the large portion of the network), on the other hand, are dramatically affected by the thermal treatment. The initial enhancement in melt elasticity (increase in the G’ parameter at 0.1 rad/s), brought about the presence of the silica, gradually decreases to levels below the values exhibited by the pristine ENR system after about 600 s, levelling off from about 3500 s onwards. A reduction in viscous component (decrease in the G” parameter at low frequency, 0.1 rad/s) resulting from the presence of silica is observed right from the beginning of the thermal treatment, reaching a plateau at around 3500 s. Apart from the peculiar behavior observed in the β’’curve over the interval around 1200–3600 s of the thermal treatment, the lowering of the β’ and β’’ to values below 1 (i.e., below the zero line on log scale) is indicative of a predomination of chain scission reactions over cross-linking within the ENR matrix, resulting from the catalytic effect of the silica particles throughout the thermal history of the sample.

## 4. Conclusions

In this work, time-resolved mechanical spectroscopy (TRMS) was employed to study the rheological response on an ENR-based composite containing 10 wt.% of silica particles, within both the linear and non-linear rheological regime. The data have revealed a remarkable effect of the silica particles on the macromolecular dynamics of the ENR matrix, which has been attributed to the formation of a matrix/polymer interphase exhibiting highly impeded relaxations. At the same time, the analysis of the storage and loss modulus curves obtained through TRMS sweeps for both the ENR + SiO_2_ composite and the pristine ENR has shown that the silica particles exhibit a catalytic effect on the degradation of the ENR matrix. This effect was evidenced from an increase in gelation time, as well as through the analysis of a purposely derived factor that takes into account the relative changes associate with the variations of the storage and loss modulus during the thermal treatment. The obtained data provide useful insights for the applications of silica reinforced ENR and particularly on the role of silane coupling agents, which may extend beyond the enhancement of interfacial adhesion to include that of a stabilizing agent.

## Data Availability

The data presented in this study are available on request from the corresponding author.
